# Health and intention to leave the profession of nursing - which individual, social and organisational resources buffer the impact of quantitative demands? A cross-sectional study

**DOI:** 10.1186/s12904-020-00589-y

**Published:** 2020-06-17

**Authors:** Elisabeth Diehl, Sandra Rieger, Stephan Letzel, Anja Schablon, Albert Nienhaus, Luis Carlos Escobar Pinzon, Pavel Dietz

**Affiliations:** 1 Institute of Occupational, Social and Environmental Medicine, University Medical Center of the Johannes Gutenberg University Mainz, Obere Zahlbacher Str, 67 55131 Mainz, Germany; 2 Institute for Health Services Research in Dermatology and Nursing (IVDP), University Medical Centre Hamburg-Eppendorf, Hamburg, Germany; 3 Department for Occupational Medicine, Hazardous Substances and Health Science, Institution for Accident Insurance and Prevention in the Health and Welfare Services (BGW), Hamburg, Germany; 4 Federal Institute for Occupational Safety and Health (BAuA), Berlin, Germany

**Keywords:** Nurses, Palliative care, Stress, Strain, Burnout, Moderator analyse, Prevention

## Abstract

**Background:**

The aim of this study was to analyse the buffering effect of individual, social and organisational resources on health and intention to leave the profession in the context of burden due to quantitative job demands.

**Methods:**

In 2017, a cross-sectional survey was carried out anonymously among nurses in palliative care in Germany. One thousand three hundred sixteen nurses responded to the questionnaire (response rate 38.7%), which contained, amongst others, questions from the German version of the Copenhagen Psychosocial Questionnaire (COPSOQ). Moderator analyses were conducted to investigate the buffering effect of different resources on health (‘self-rated health’ and ‘burnout’) and ‘intention to leave’ in the context of quantitative demands.

**Results:**

‘Self-rated health’ was significantly buffered by the resources ‘recognition through salary’ (*p* = 0.001) and ‘good working team’ (*p* = 0.004). Additionally, buffering effects of the resources ‘workplace commitment’ and ‘good working team’ on ‘burnout’ (*p* = 0.001 and *p* = 0.006, respectively) as well as of the resources ‘degree of freedom’, ‘meeting relatives after death of patients’, ‘recognition from supervisor’ and ‘possibilities for development’ on ‘intention to leave’ (*p* = 0.014, *p* = 0.012, *p* = 0.007 and *p* = 0.036, respectively) were observed.

**Conclusions:**

The results of our study can be used to develop and implement job (re) design interventions with the goal of reducing the risk of burnout and enhancing job satisfaction among nurses in palliative care. This includes for example adequate payment, communication training and team activities or team events to strengthen the team as well as the implementation of some rituals (such as meeting relatives after the death of patients). As our study was exploratory, the results should be confirmed in further studies.

## Background

In Germany just as in other countries, the number of elderly people is continuously increasing [1]. Patients with incurable and life-threatening diseases have a legal entitlement to specialist palliative care in the context of specialist outpatient palliative care services (SAPV), in inpatient hospices or in palliative care units [2]. In future, not only persons with cancer but also persons with non-oncological diseases such as chronic heart failure, chronic obstructive pulmonary disease as well as multimorbid patients will benefit from palliative care [3]. This creates new challenges for the labour market and the health care system. According to the Federal Statistical Office in Germany, the number of people in need of nursing will increase from 3.4 million to 5.4 million by the year 2050 [4]. Because nurses play a central role in delivering health care and their own health may have an effect on the quality of the services offered by the health care system [5], it is of particular importance to consider the working situation and health of nurses in order to improve their working situation as best as possible. Numerous national and international studies have investigated the working situation of nurses in palliative care and have studied quantitative job demands [6–10] and in particular the burden caused by death and dying [7, 8, 11, 12]. However, little attention has been given to date to the influence of the buffering effect of individual, social and organisational resources on outcomes like burnout, self-rated health and intention to leave the profession in the context of burden due to quantitative job demands.

Various studies in the field of palliative care addressed individual, social and organisational resources of nurses within the framework of occupational stress and health. However, these studies did not investiage potential buffering effects of these resources. Regarding individual resources, resilience was discussed as an important ability of nurses in decreasing occupational burden and increasing emotional wellbeing [13, 14]. Further, meaning and purpose in life, secure attachment styles, attitudes towards death [15] and empathy [16] were identified as protective factors against burnout. Organisational resources, such as job content, identification with the institution, low time pressure and a good working atmosphere [10] were identified as predictors for health stability, whereas organisational activities [17] were identified as protective factors against burnout. A study from Singapore determined that factors such as physical well-being, passion for one’s work and remembrance of patients inter alia were protective against burnout [17]. Furthermore, social resources such as social support [8, 18] as well as individual resources such as physical activity [18], self-care [19], spirituality and hobbies [17] had a protective effect on health.

Job satisfaction in the field of palliative care in Germany is high [20, 21]. The authors of this paper assume that persons who are highly satisfied with their work are not thinking about leaving their job. Thus, variable job satisfaction is associated with the likelihood of staying in the job. Results of various studies indicate that job satisfaction depends not only on stress [22], time pressure [10], schedule [23] and high workload [9] but also on resources such as a subject’s own professional skills [23], rewards, a people-oriented culture [24], the job content, identification with the institution [10], a good feeling from being able to help, a good relationship with the patient and their family [13], meeting relatives after the death of patients [21], nurses’ satisfaction with work-life balance [24] as well as doing meaningful work for others and being in a good working team [20].

The studies mentioned above considered stress, strain and resources independently using qualitative or descriptive methods or analysed the relationships between variables using correlation analyses. Studies specifically examining the buffering/moderating role of resources on health and intention to leave the profession or job satisfaction are rare. Moderator variables affect the relationship between an independent variable (such as quantitative demands) and a dependent variable (such as burnout). They can amplify or weaken the correlation between these variables [25]. A study from Spain determined that resilience moderates the effect of cynicism on health [26]. Optimism, as a personal resource, was shown to be a moderator effect on exhaustion [27]. The results of a Norwegian study suggested a buffering effect of professional commitment between job demands and emotional exhaustion [28]. Work engagement moderated the relationship between job demands and burnout, while social support was an important predictor of work engagement [29]. Further, a moderating effect of emotional intelligence between work demands and burnout was identified [30].

Based on the knowledge gap addressed above, we have analysed the working situation of nurses using the Rudow Stress-Strain-Resources model [31]. According to this model, individual, social and organisational resources of a person buffer/moderate the negative effects of job demands (stress) on, for example, health (strain).

The aim of this study was to determine which resources buffer the impact of quantitative job demands on health (‘self-rated health’ and ‘burnout’) and ‘intention to leave the profession’.

## Methods

### Study design

In 2017, an anonymous cross-sectional survey was performed among nurses in the field of specialised palliative care in Germany. In Germany, palliative care is divided into general and specialised palliative care. Specialised palliative care includes palliative care units in hospitals, inpatient hospices and the SAPV. Palliative care units are independent specialist institutions integrated within a hospital. Palliative care units stabilize or improve the condition of patients in order to discharge them, if possible, to their own homes. Inpatient hospices are independent facilities which ensure palliative care for people with incurable, life-threatening diseases as well as a dignified death where such care cannot be provided in the home environment. SAPV-teams should enable palliative care and a dignified death in familiar surroundings [2]. Since there was neither information on the number of specialist palliative care institutions nor on the number of nurses working in this field, an internet search was perfomed by the research team, in which 950 palliative care facilities from all over Germany were identified, of which 358 were SAPV institutions, 343 were palliative care units and 249 were inpatient hospices. From the identified number of facilities, an institution-related random sample was drawn. Ultimately, 532 palliative care facilities were included of which 246 (46.2%) were willing to participate in the study. These palliative care facilities reported the number of nurses, geriatric nurses, nursing assistants and nurses in training to the study team and whether they would be willing to participate either via a paper-and-pencil questionnaire (with a pre-franked envelope) or an online survey. A total of 3539 questionnaires were sent out (2773 paper-and-pencil and 766 online questionnaires): 1366 to nurses in hospices, 1178 to nurses in SAPV institutions and 995 to nurses in palliative care units.

### Questionnaire

The questionnaire contained i) questions on sociodemographic information as well as ii) characteristics on current profession. iii) Scales of the German version of the Copenhagen Psychosocial Questionnaire (COPSOQ) – version 2016 were used to measure occupational stress (scale ‘quantitative demands’), health, intention to leave the profession and various resources. Further, iv) a resilience questionnaire [RS-13] consisting of 13 items was used to measure resilience. The scale focuses on attributes of the core concept, such as emotional stability, optimism, and vitality [32]. Additionally, v) single questions regarding resources were added which were frequently reported by nurses of specialised palliative care as being helpful in dealing with the demands of the work in the pilot study. The pilot study was carried out in 2015. First, guided interviews with different professionals in the field of palliative care were conducted and analysed [33]. Based on the results of these interviews, a questionnaire was developed which focused on nurses working in palliative care. This questionnaire included, among other things, questions about resources. The questionnaire was first, preliminary tested in a sample of 16 nurses working in palliative care and then used in a sample of nurses working in specialised palliative care institutions in Rhineland-Palatinate [21, 34]. Table 1 presents an overview of the sources and variables reported in this paper. It took about 20 min to fill in the questionnaire. The COPSOQ is of generic usability for different occupational groups, this particulary involves nursing care [35–40]. The assessment of the reliability, generalisability and validity of the single COPSOQ scales showed medium to good measuring qualities for the majority of the scales (i.e. Cronbach’s alpha mostly > 0.70) in the German COPSOQ validation study [41]. The RS-13 is the short German version of the 25-item Resilience Scale which was developed by Wagnild and Young [42] and has been validated in representative clinical and nonclinical samples (Cronbach’s alpha = 0.90) [32, 43].

**Table 1:** Sources and variables of the questionnaire

Measure	Concstruct	Variable	# of items	Example and interpretation
**COPSOQ**				
	**workload**	scale ‘quantitative demands’	*4*	*Do you have to work very fast?*
	**health**	scale ‘self-rated health’	*1*	*Your state of health: If you evaluate the best conceivable state of health at 10 points and the worst at 0 points: How many points do you then give to your present state of health? Please put a cross by the corresponding number.*
		scale ‘burnout’	*6*	*How often do you feel emotionally exhausted?*
	**intention to leave the profession**	scale ‘intention to leave the profession’	*1*	*How often in the course of the past year have you thought about giving up your profession?*
				response categories: never, a few times a month, once or twice a week, three to five times a week and every day; for the logistic regression analysis the “intent to leave the nursing profession” variable was dichotomized: ‘never’ vs. ‘at least one time’ (a few times a month, once or twice a week, three to five times a week, every day)
	**resources**			
		scale ‘influence at work’	*4*	*Do you have any influence on what you do at work?*
		scale ‘degree of freedom at work’	*4*	*Can you decide when to take a break?*
		scale ‘possibilities for development’	*4*	*Do you have the possibility of learning new things through your work?*
		scale ‘meaning of work’	*3*	*Do you feel that the work you do is important?*
		scale ‘workplace commitment’	*4*	*Do you enjoy telling others about your place of work?*
**Resilience questionnaire RS-13**				
	**resilience**	resilience	*13*	*I can accept it when not all people like me.*
				total score range from 13 to 91, low resilience, score 67–72: moderate resilience, score 73–91: high resilience
**Pilot study**				
	**single question**	meaningfulness of work	*1*	*How much do the following help you to handle the workload?*
				Not helpful, little helpful, quite helpful, very helpful
	**single questions**	meeting the relatives after death of patients, family, friends, professional attitude/dissociation, positive thinking, hobbies, self-reflection, self-care, physical activity, religiosity/spirituality	*1 question for each variable*	*How much do the following help you to handle the workload?*
				Not helpful, little helpful, quite helpful, very helpful
	**single questions**	recognition from supervisor, recognition from patients and relatives, recognition from colleagues, recognition through social context, recognition through salary, good working team, help and support from colleagues in emergencies	*1 question for each variable*	*Do you receive recognition for your work from …*?
				Do not agree at all, rather disagree, somewhat agree, fully agree

COPSOQ-scales have a score from 0 to 100, high = positive, only regarding the scales ‘quantitative demands’ and ‘burnout’, high = negative

### Statistical analysis

The data of the paper-and-pencil and online questionnaire were merged. Data cleaning and plausibility checks were performed (e.g. questionnaires without specification of the working field were delated). Scales were formed according to the COPSOQ guidelines [44]. In general, COPSOQ items have a 5-point Likert format (for example scale ‘quantitative demands: 1 = always, 5 = never), which are transformed to a 0 to 100 scale. This transformation is a standardized procedure and conforms to the German COPSOQ validation study [34]. The scale score is calculated as the mean of the items for each scale. If at least 50% of the items of a scale were answered, the scale value was calculated as the average of the items answered. If less than 50% of the items of a scale were answered, the scale value was regarded as missing. Cronbach’s alpha was used to assess the internal consistency of the scales. A Cronbach’s alpha > 0.7 was regarded as acceptable [25]. The 13 items of the resilience questionnaire RS-13 are based on a 7-point Likert format (1 = I do not agree, 7 = I totally agree). The score of the resilience questionnaire (only computed when all 13 items had valid answers) range from 13 to 91. According to the literature, the results of the resilience questionnaire were grouped (low resilience = score 13–66, moderate resilience = score 67–72 and high resilience = score 73–91) [32]. The answer categories of the single questions about resources were dichotomised (e.g. not helpful/little helpful vs. quite helpful/very helpful). The study was conceptualised as an exploratory study, so that the *p*-values merely enable the recognition of any statistically noteworthy findings [45]. The results of univariate analyses are presented in terms of absolute and relative frequencies. Furthermore, we present means (M) and standard deviations (SD). Bivariate statistics were used to infer important variables (covariates and moderators) for the moderator analysis, where the scale ‘quantitative demands’ were treated as independent variable (see Table 2 and additional Tables 1, 2 and 3). The scales ‘intention to leave the profession’ and ‘workplace commitment’ did not fulfil all conditions for linear regression analysis and were therefore treated as categorical variables for the analysis [25]. The scale ‘intention to leave the profession’ had the answer categories ‘never’, ‘a few times a month’, ‘once or twice a week’, ‘three to five times a week’ and ‘every day’. This was divided into the categorical answer groups ‘never’ vs. ‘at least one time in the last year’.

**Table 2:** Correlations of continuous variables

	1.	2.	3.	4.	5.	6.	7.	8.
**independent variable**								
1. quantitative demands	1							
**dependent variables**								
2. burnout	.442**	1						
3. self-rated health	−.283**	−.554**	1					
**resources**								
4. workplace commitment	−.084^**^	−.119**	.090**	1				
5. degree of freedom	−.329^**^	−.248**	.185**	.165^**^	1			
6. possibilities for development	−.044	−.151**	.150**	.287^**^	.303^**^	1		
7. influence at work	−.211^**^	−.215**	.131**	.219^**^	.544^**^	.398^**^	1	
8. meaning of work	−.224^**^	−.257**	.212**	.411^**^	.236^**^	.534^**^	.275^**^	1

Pearson correlation, ** *p* ≤ 0.01

The program *PROCESS* developed by Andrew F. Hayes was used in order to determine whether the relationship between two variables (‘quantitative demands’ and ‘self-rated health’ or ‘burnout’ or ‘intention to leave the profession’) depends on the value of a third variable (resource) [25, 46]. Using the PROCESS tool has several advantages over using the normal regression tools in SPSS: (1) it centers predictors; (2) it computes the interaction term automatically and (3) it does simple slopes analysis [25] which makes it easier to understand the interaction effect.

For the analyses of ‘self-rated health’ and ‘burnout’ as dependent variables, linear regression methods of the PROCESS program were used. In a first step, two variables were included: ‘quantitative demands’ and one resource (per model). These variables accounted for a significant amount of variance in for example ‘self-rated health’. In a second step, an interaction term between ‘quantitative demands’ and one resource (per model) was created and added to the regression model. If the model with the interaction term accounted for significantly more variance than model 1 (R^2^), this indicated that there was potentially moderation between ‘quantitative demands’ and the resource on ‘self-rated health’ [47]. For the analyses of ‘intention to leave the profession’, logistic regression methods of the PROCESS program were used (yes (1) vs. no (0)). Simple effect coefficients were used to calculate the odds ratios with 95% confidence intervals [CI]. To probe the interaction, three values of the continuous resource variables (within +/− 1 SD of the mean) or the value of the characteristics of the categorical resource variables were computed. The moderator analysis was performed using centred terms and were adjusted for age, sex, working area and extent of employment as well as for further covariates if they were significant (*p* ≤ 0.05) within the bivariate analyse. If moderation was observed, the interaction of the variables was plotted [25, 46].

Analyses were performed using SPSS version 23.5 and PROCESS for SPSS (Version 2.16.3) for the moderator analysis.

## Results

### Descriptive analyses

One thousand three hundred seventy-one nurses filled in the questionnaire resulting in an overall response rate of 38.7%. Of these, 1171 prepared the paper-and-pencil version (response rate 42.2%) and 200 the online version (response rate 26.1%). Palliative care units had the overall highest response rate (44.1%), followed by hospices (39.9%) and SAPVs (29.0%). Fifty-five questionnaires were excluded from data analysis (*n* = 45: no assignment to a SAPV, palliative care unit or hospice possible) resulting in a total number of 1316 (96.0%) questionnaires included for the analysis.

One thousand one hundred nineteen of the nurses (87.3%) were female and 582 (45.1%) were 50 years old or older. Prior to the year 2020, the vocational German courses for nursing care included nurses, geriatric nurses and paediatric nurses. Further, it was possible to complete a University degree to become a nurse. Nursing assistants acts as links between senior nurses and the patient, and help with the health care of patients under the supervision of a senior nurse. In the present study, 835 (64.8%) were nurses, 221 (17.2%) were nursing assistants or in training, 136 (10.6%) were geriatric nurses and 96 (7.5%) nurses were university graduates. 538 (40.9%) of the nurses worked in hospices, 441 (33.5%) in palliative care units and 337 (25.6%) in SAPV institutions. About half of the nurses (*n* = 616; 47.4%) had already been working between 16 and 30 years as nurses. 233 (17.9%) served in an advisory function only, meaning that that they did not engage in any practical nursing activity. 575 (44.4%) had a full-time job. The total list of nurses’ characteristics is presented in Table 3.

**Table 3:** Socio-demographic data (*n* = 1316)

Variable		Number	Percent
sex	male	163	12.7
	female	1119	87.3
age	≤ 39 years	342	26.5
	40–49 years	366	28.4
	≥ 50 years	582	45.1
marital status	single	316	24.8
	married	722	56.8
	divorced	207	16.3
	widowed	27	2.1
children in household	no	708	55.5
	yes	567	44.5
education	without a school-leaving qualification/ secondary school leaving certificate/ other qualification	72	5.6
	intermediate school-leaving certificate	674	52.5
	qualification for university entrance	538	41.9
grade	nursing assistant/ in training	221	17.2
	geriatric nurse	136	10.6
	nurse	835	64.8
	university graduate	96	7.5
working area	SAPV	337	25.6
	hospice	538	40.9
	palliative unit	441	33.5
duration of nursing activities	0–15 years	401	30.5
	16–30 years	616	47.4
	31–50 years	283	21.8
exercise of nursing procedures^a^	no	233	17.9
	yes	1071	82.1
extent of employment	full-time job	575	44.4
	≥ 76%	183	14.1
	51–75%	316	24.4
	≤ 50%	221	17.1
fund	publicly-owned	338	26.4
	private	209	16.4
	independent	731	57.2

Shown are valid percentages; missing values: sex (*n* = 34), age (*n* = 26), marital status (*n* = 44), children in household (*n* = 41), graduation (*n* = 32), education (*n* = 28), duration of nursing activities (*n* = 16), extent of employment (*n* = 21), fund (*n* = 38); ^a^Nurses working in SAPV institutions can work only in an advisory function, this means they, for example, coordinate the outpatient care with SAPV care or consultant the family members. These nurses do not patient care in traditional sense [48]

Table 4 presents the mean scores and standard deviations for the COPSOQ scales ‘quantitative demands’, ‘self-rated health’, ‘burnout’ as well as ‘intention to leave the profession’. Further, the resource scales ‘influence at work’, ‘degree of freedom at work’, ‘possibilities for development’, ‘meaning of work’ and ‘workplace commitment’ are presented. Additionally, the number of scale items and the internal consistency is listed. All scales in this study achieve satisfactory values of internal consistency. Only the scale ‘degree of freedom at work’ shows a lower value of 0.687.

**Table 4:** Means and standard deviations of the COPSOQ scales

Variable	number of items	Cronbach’s α	n	M (SD)^a^
quantitative demands	4	0.798	1313	42.67 (18.47)
self-rated health	1	–	1303	72.86 (16.94)
burnout	6	0.907	1311	41.43 (17.61)
intention to leave the profession	1	–	1311	12.87 (19.21)
influence at work	4	0.728	1293	48.97 (19.86)
degree of freedom at work	4	0.687	1291	52.72 (20.46)
possibilities for development	4	0.704	1297	76.22 (14.98)
meaning of work	3	0.827	1296	88.32 (13.34)
workplace commitment	4	0.711	1295	60.79 (18.72)

^a^M = mean; *SD* standard deviation

569 (43.8%) of the nurses reported that ‘meeting relatives after the death of patients’ was helpful to deal with the demands of their work. 878 (68.0%) of the nurses agreed with the statement that they received ‘recognition of their work from the supervisor’ and 348 (26.8%) through ‘the salary’. One thousand ninety-nine nurses (84.4%) stated that they could not do this job without a ‘good working team’. 362 (28.8%) of the nurses had a low, 267 (21.3%) a moderate and 623 (49.8%) a high ‘resilience’.

### Moderator analyses

#### Self-rated health

There was a negative and significant association between ‘self-rated health’ and ‘quantitative demands’ (b = − 0.24, SE = 0.026, *p* < 0.001) and a positive association between ‘self-rated health’ and ‘recognition through salary’ (b = 6.54, SE = 1.063, *p* < 0.001). The interaction term of ‘quantitative demands’ and the resource ‘recognition through salary’ accounted for significantly more variance in ‘self-rated health’ than a model without an interaction term (∆R^2^ = 0.007, b = 0.18, SE = 0.055, *p* = 0.001). Regarding the resource ‘good working team’, the variables ‘self-rated health’ and ‘quantitative demands’ were negative and significantly associated (b = − 0.24, SE = 0.026, p < 0.001) and the variables ‘self-rated health’ and ‘good working team’ were not associated (*p* = 0.224). The interaction term of ‘quantitative work demands’ and the resource ‘good working team’ accounted for significantly more variance in ‘self-rated health’, than a model without an interaction term (∆R^2^ = 0.006). The resource ‘good working team’ moderated the impact of ‘quantitative work demands’ on ‘self-rated health’ (b = 0.35, SE = 0.122, *p* = 0.004) (see Additional Table 4).

**Fig. 1:**
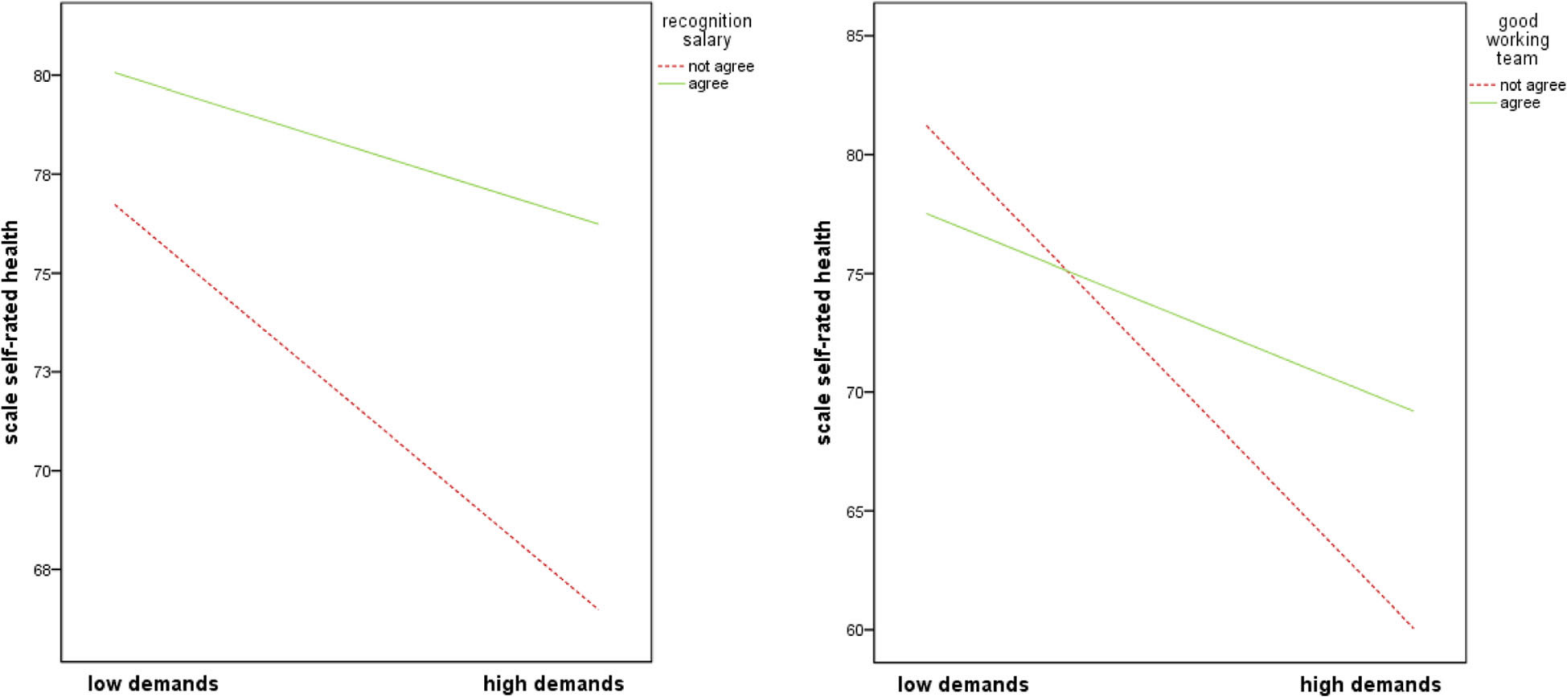
Interaction between ‘quantitative demands’ and resources in predicting ‘self-rated health’

Examination of the interaction plots showed that at low ‘quantitative demands’, ‘self-rated health’ was quite similar for the nurses which affirmed or denied having the resources. Irrespective of whether or not the resources were helpful in dealing with the quantitative demands, higher quantitative demands had a negative effect on ‘self-rated health’, but when demands increased, nurses who affirmed having the resources stated a better ‘self-rated health’ than nurses who denied having it. The resources ‘recognition through salary’ and ‘good working team’ worked as moderators regarding ‘self-rated health’ (Fig. 1).

#### Burnout

There was a positive and significant association between ‘burnout’ and ‘quantitative demands’ (b = 0.42, SE = 0.026, *p* < 0.001) and a negative and significant association between ‘burnout’ and ‘workplace commitment’ (b = − 2.60, SE = 0.968, *p* = 0.007). The interaction term of ‘quantitative demands’ and the resource ‘workplace commitment’ accounted for significantly more variance in ‘burnout’ than a model without an interaction term (∆R^2^ = 0.007, b = − 0.17, SE = 0.052, *p* = 0.001). Regarding the resource ‘good working team’, the variables ‘burnout’ and ‘quantitative demands’ were positively and significantly associated (b = 0.43, SE = 0.026, p < 0.001), while the variables ‘burnout’ and ‘good working team’ were negatively and significantly associated (b = − 4.75, SE = 2.182, *p* = 0.030). The interaction term of ‘quantitative demands’ and the resource ‘good working team’ accounted for significantly more variance in ‘burnout’, than a model without an interaction term (∆R^2^ = 0.005, b = − 0.33, SE = 0.118, *p* = 0.006) (see Additional Table 5).

**Fig. 2:**
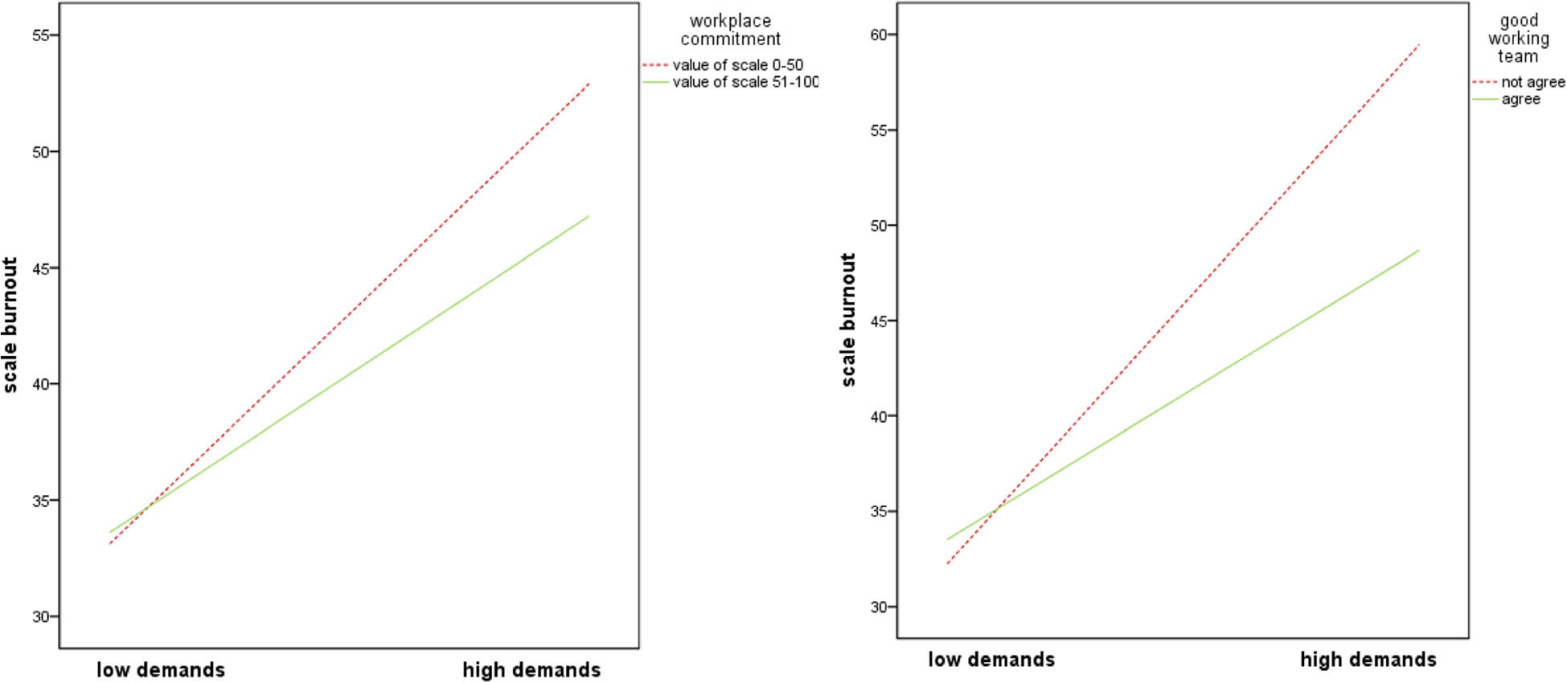
Interaction between ‘quantitative demands’ and resources in predicting ‘burnout’

Examination of the interaction plot demonstrated that the resources ‘workplace commitment’ and ‘good working team’ influenced the impact of ‘quantitative demands’ on ‘burnout’. At low ‘quantitative demands’ the ‘burnout’ was quite similar for nurses with and without the resources and higher ‘quantitative demands’ had a negative effect on ‘burnout’. But when the demands increased, nurses who affirmed having the resources stated a lower burnout level than nurses who denied having it (Fig. 2).

#### Intention to leave the profession

The results indicated a significant interaction of the variables ‘quantitative demands’ and ‘degree of freedom’ regarding ‘intention to leave the profession’ (b = − 0.0005, SE = 0.0002, OR = 0.9995 [0.9992, 0.9999, *p* = 0.014, see Additional Table 6). At all levels of ‘degree of freedom’, higher ‘quantitative demands’ were associated with a significant increase in odds of ‘intention to leave the profession’ but the highest increase was associated with the lowest ‘degree of freedom’ (below mean: b = 0.03, SE = 0.006, OR = 1.03 [1.02, 1.05], *p* < 0.001; at the mean: b = 0.02, SE = 0.004, OR = 1.02 [1.02, 1.03], *p* < 0.001; above mean: b = 0.01, SE = 0.006, OR = 1.01 [1.004, 1.03], *p* < 0.009.). Regarding the resource ‘meeting relatives after death of patients’ a significant interaction of this variable and ‘quantitative demands’ regarding ‘intention to leave the profession’ (b = − 0.02, SE = 0.007, OR = 0.98 [0.97, 0.996], *p* = 0.012) was assessed (see Additional Table 7). Both nurses who reported that ‘meeting relatives after death of patients’ would be helpful and nurses denying this had a significant increase in odds of ‘intention to leave the profession’ when the quantitative demands increased but this was higher in the group which denied this (no ‘meeting relatives after death of patients’: b = 0.04, SE = 0.005, OR = 1.04 [1.03, 1.01], *p* < 0.001), ‘meeting relatives after death of patients’: b = 0.017, SE = 0.006, OR = 1.02 [1.01, 1.03], *p* = 0.002). Moreover, a significant interaction of the variables ‘quantitative demands’ and ‘recognition from supervisor’ regarding ‘intention to leave the profession’ (b = − 0.02, SE = 0.008, OR = 0.98 [0.96, 0.99], *p* = 0.007) was observed (see Additional Table 8). Both nurses who affirmed having ‘recognition from supervisor’ as well as nurses denying having this had a significant increase in the odds of ‘intention to leave the profession’ when the demands increased but this was higher by the nurses who denied this (no ‘recognition from supervisor’: b = 0.04, SE = 0.007, OR = 1.04 [1.02, 1.05], *p* < 0.001, ‘recognition from supervisor’: b = 0.02, SE = 0.005, OR = 1.02 [1.01, 1.02], p = 0.002). The interaction of the variables ‘quantitative demands’ and the scale ‘possibilities for development’ regarding ‘intention to leave the profession’ was significant (b = − 0.0005, SE = 0.0002, OR = 0.9995 [0.9992, 1.0], *p* = 0.036, see Additional Table 9). At all levels of ‘possibilities for development’, higher ‘quantitative demands’ were associated with a significant increase in odds of ‘intention to leave the profession’ but the highest increase was associated with the lowest ‘possibilities for development’ (below mean: b = 0.04, SE = 0.006, OR = 1.04 [1.03, 1.05]; at the mean: b = 0.03, SE = 0.039, OR = 1.03 [1.02, 1.04]; above mean: b = 0.02, SE = 0.005, OR = 1.02 [1.01, 1.03], p < 0.001).

**Fig. 3:**
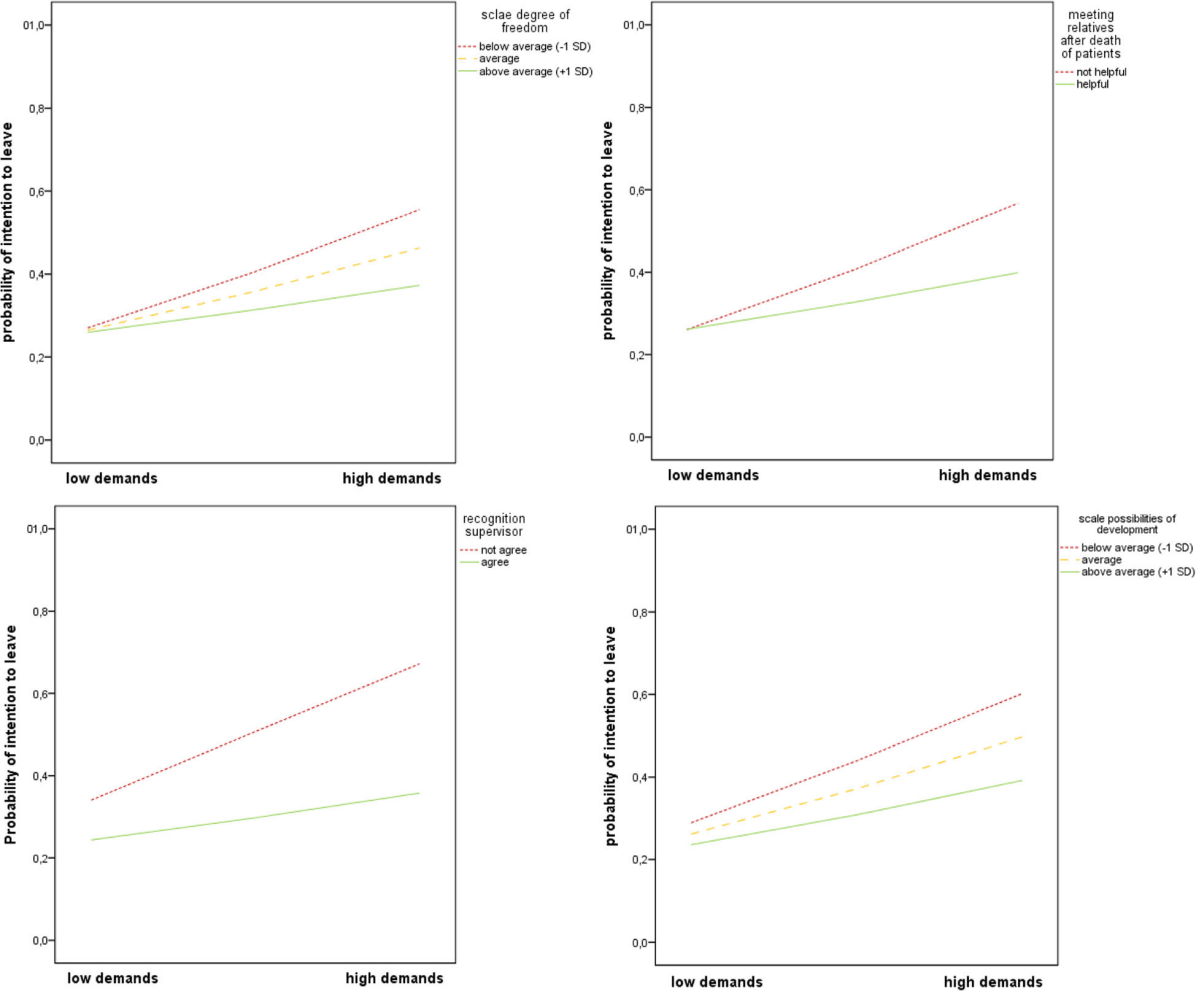
Interaction between ‘quantitative demands’ and resources in predicting the probability of ‘intention to leave’

Examination of the interaction plot revealed that the resources ‘degree of freedom’, ‘meeting relatives after death of patients’, ‘recognition from supervisor’ and ‘possibilities for development’ influenced the impact of ‘quantitative demands’ on ‘intention to leave the profession’. Irrespective of whether or not the resources were helpful in dealing with the quantitative demands, higher quantitative demands had a negative effect on ‘intention to leave the profession’, but when the demands increased, nurses who affirmed having the resources stated a lower ‘intention to leave the profession’ than nurses who denied having it (Fig. 3).

## Discussion

According to the Rudow Stress-Strain-Resources model outlined in the introduction, a buffering effect of the resources ‘recognition through salary’ and ‘good working team’ on ‘self-rated health’ was observed. Furher, the buffering effect of the resources ‘workplace commitment’ and ‘good working team’ on ‘burnout’ was evaluated and a buffering effect of the resources ‘degree of freedom’, ‘meeting relatives after death of patients’, ‘recognition from the supervisor’ and ‘possibilities for development’ concerning ‘intention to leave the profession’ was assessed.

The results of the study show that the team is a crucial resource concerning both health and burnout. This tallies with the results of other studies which identified the team as an essential resource in the field of palliative care [10, 11, 34, 49, 50]. The feeling of recognition of the work carried out as valuable and sensible present a protective factor [24, 51]. In the present study recognition through the salary has a buffering effect on health and recognition from the supervisor has a buffering effect on job satisfaction. Moreover, the results confirm that the degree of freedom (with respect to working time, breaks or vacation) and development possibilities (such as education and training) are associated with job satisfaction [52–54] . We can also confirm the results of a study in Serbia, where a buffering effect of workplace commitment regarding burnout was found [55]. However, a study from Estonia found no association between workplace commitment and burnout [52]. Meeting of the relatives after the death of patients is a very specific aspect which receives hardly any attention in the literature. We discussed this aspect within the paper of a previously published pilot study for the first time as a resource for nurses in palliative care [21]. The results of the present study show that contact with relatives after the death of patients is a very important resource. However, this aspect needs further scientific investigation.

Using the validated COPSOQ as a survey instrument provided the possibility of using comparative data from other studies in the field of care in Germany. According to Nübling, a difference of at least 5 points in the mean value of a COPSOQ scale demonstrates a relevant difference between groups [44, 56]. This study was the first survey in the field of the specialist palliative care, thus the comparative data was from the general health care system in Germany. The ‘quantitative demands’ reported by the nurses in this study are lower than those reported by nurses of the general health care system [35, 36]. Concerning ‘self-rated health’ there were no differences to other studies [36, 37]. One study presented nearly identical results regarding ‘burnout’ [36] whereas another study identified higher values [37]. The scale ‘intention to leave the profession’ had a mean of 13, which is lower compared to other studies [38, 39]. Regarding the scale ‘influence at work’, which indicates how much self-determined work is possible, the nurses reported a value that was much higher than the values ascertained in other studies [35, 37, 39, 40]. The nurses in this survey reported also a higher value concerning the scale ‘degree of freedom’ than nurses in other fields [37, 40]. The mean of the scale ‘possibilities for development’ was lower [37, 40] as well as higher in the comparative data [35], while the nurses in the latter study were relatively young and therefore may have had more opportunities for development. The mean of the scale ‘meaning of work’ was lower in comparable studies [36, 37, 39]. Nurses in palliative care valued ‘workplace commitment’ higher than nurses of comparative studies [36, 37, 39].

The reporting of lower ‘quantitative demands’ could be explained through the system. Nurses in specialist palliative care in Germany had fewer patients to care for than nurses in other fields [57]. The health of the palliative care nurses was not different to other fields, but they had less ‘intention to leave the profession’ compared to nurses of other fields. Concerning the resources, the resources ‘influence at work’, ‘degree of freedom’ and ‘meaning of work’ were significantly higher. That hardly comes as a surprise because these findings were in line with the results presented in this study. Lower quantitative demands enabled the nurses to spend more time with patients and engage in care activities as they found appropriate and sensible. This was associated with a feeling of more influence over their work and a degree of freedom.

In the light of demographic development the results of the intention of leaving the job are very important. Palliative care nurses seemed to be more satisfied than nurses of other fields. This could be explained by the fact that on the one hand, they reported fewer ‘quantitative demands’, while on the other hand, they listed numerous resources. Concerning the ‘degree of freedom’ and the ‘possibilities for development’, a buffering effect regarding the ‘intention to leave the profession’ was assessed. Furthermore, a buffering effect of the resources ‘recognition from the supervisor’ and ‘recognition through the salary’, ‘good working team’, ‘workplace commitment’ and ‘meeting relatives after the death of patients’ was observed.

The results underline current efforts of the Federal Government in Germany with the Nursing Workforce Strengthening Act (Pflegepersonal-Stärkungs-Gesetz (PpSG)) concerning an improvement of working conditions, for example regarding the salary and personnel requirements. Furthermore, specific courses of action should be discussed, including strengthening of the team in order to build and maintain good relationships, recognition from supervisors, a higher degree of freedom for nurses as well as the implementation of some new procedures (like meeting the relatives after the death of patients). Additionally, future studies should review specialist palliative care as the best practice example for nursing care in Germany.

### Limitations

There was no information available about the total population of nurses working in specialised palliative care. The present study therefore focused firstly on palliative care facilities. Only the participating facilities reported the number of staff members. Therefore, despite the participation of near 1400 nurses of the palliative care in Germany, the potential for selection bias has to be discussed. A comparison with participants and non participants was not feasible. It was possible that the institutions and nurses experiencing the highest burdens in particular were less inclined to engage in a time-consuming survey, so that the results in particular of the demands were underestimated. Further, our research aim was to investigate the resources and demands of persons working in palliative care. Therefore we included students and trainees in the sample, because they already work in palliative care. It should be born in mind, that students and trainees, particularly if they are at an early stage in their training, are maybe not comparable in terms of stresses and strains and resources to the group of nurses with many years of experience. Due to the cross-sectional design of the survey, it was not possible to establish causal relationships.

## Conclusions

The results of our study can be used to develop and implement job (re) design interventions with the goal of reducing the risk of burnout and enhancing job satisfaction in palliative care nurses. This includes for example adequate payment, communication training and team activities or team events to strengthen the team as well as the implementation of some new procedures (like meeting relatives after the death of patients). As our study was exploratory, the results should be veriefied by further studies.

## Supplementary information

**Additional file 1: Table 1**. Associations between the scale ‘quantitative demands’ and categorical variables.

**Additional file 2: Table 2**. Associations between the scale ‘burnout’ and covariates.

**Additional file 3: Table 3**. Associations between ‘intention to leave the profession’ and covariates.

**Additional file 4: Table 4**. Coefficients of the moderated regression model for ‘self-rated health’

**Additional file 5: Table 5**. Coefficients of the moderated regression model for ‘burnout’

**Additional file 6: Table 6**. Coefficients of the moderated logistic regression of ‘intention to leave’ and resource ‘degree of freedom’.

**Additional file 7: Table 7**. Coefficients of the moderated logistic regression of ‘intention to leave’ and ‘meeting relatives after death’.

**Additional file 8: Table 8**. Coefficients of the moderated logistic regression of ‘intention to leave’ and resource ‘recognition from supervisor’.

**Additional file 9: Table 9**. Coefficients of the moderated logistic regression of ‘intention to leave’ and resource ‘possibilities for development’.

## Data Availability

The whole data set is available at the University Medical Centre of the University of Mainz, Department of Occupational, Social and Environmental Medicine. Contact: elidiehl@uni-mainz.de

## Abbreviations

COPSOQ: Copenhagen Psychosocial Questionnaire
SAPV: Specialist outpatient palliative care services
RS-13: Resilience questionnaire-13
M: Means
SD: Standard deviations
b: Regression coefficient
SE: Standard error
OR: Odds ratio
CI: Confidence interval
