# Supplementary material for: Health and intention to leave the profession of nursing - which individual, social and organisational resources buffer the impact of quantitative demands? A cross-sectional study
Source: BMC Palliat Care. 2020 Jun 17;19:83. doi: 10.1186/s12904-020-00589-y (PMC7298824; doi:10.1186/s12904-020-00589-y)
Supplement: Supplementary file 1 — Additional file 1: Table 1. Associations between the scale ‘quantitative demands’ and categorical variables. [file 12904_2020_589_MOESM1_ESM.docx]

Additional Table 1: Associations between the scale ‘quantitative demands’ and categorical variables

| **Variables** | **Characteristics** | **N (%)** | **M** | **SD** | **p** |
| --- | --- | --- | --- | --- | --- |
| **dependent variable** |  |  |  |  |  |
| intention to leave | never | 811 (61.9) | 39.25 | 17.95 | < 0.001** |
|  | at least one time | 500 (38.1) | 48.20 | 18.05 |  |
| **resources** |  |  |  |  |  |
| meaningfulness of work | not/little helpful | 110 (8.5) | 46.48 | 18.71 | 0.020* |
|  | quite/very helpful | 1,187 (91.5) | 42.19 | 18.44 |  |
| meeting the relatives | not/little helpful | 729 (56.2) | 43.02 | 18.45 | 0.320 |
|  | quite/very helpful | 569 (43.8) | 41.99 | 18.56 |  |
| recognition from supervisor | do not agree/rather disagree | 414 (32.0) | 49.04 | 17.81 | < 0.001** |
|  | somewhat agree/fully agree | 878 (68.0) | 39.51 | 17.91 |  |
| recognition from patients and relatives | do not agree/rather disagree | 22 (1.7) | 49.72 | 19.04 | 0.066 |
|  | somewhat agree/fully agree | 1,284 (98.3) | 42.43 | 18.38 |  |
| recognition from colleagues | do not agree/rather disagree | 119 (9.1) | 49.39 | 19.77 | < 0.001** |
|  | somewhat agree/fully agree | 1,182 (90.9) | 41.85 | 18.14 |  |
| recognition through social context | do not agree/rather disagree | 139 (10.7) | 45.65 | 17.88 | 0.036* |
|  | somewhat agree/fully agree | 1,158 (89.3) | 42.18 | 18.46 |  |
| recognition through salary | do not agree/rather disagree | 952 (73.2) | 44.38 | 17.74 | < 0.001** |
|  | somewhat agree/fully agree | 348 (26.8) | 37.66 | 19.26 |  |
| good working team | do not agree/rather disagree | 203 (15.6) | 50.35 | 18.22 | < 0.001** |
|  | somewhat agree/fully agree | 1,099 (84.4) | 41.15 | 18.14 |  |
| help and support from colleagues in emergencies | do not agree/rather disagree | 39 (3.0) | 52.62 | 21.39 | 0.001** |
|  | somewhat agree/fully agree | 1,262 (97.0) | 42.28 | 18.28 |  |
| family | not/little helpful | 158 (12.2) | 43.31 | 17.92 | 0.617 |
|  | quite/very helpful | 1,136 (87.8) | 42.52 | 18.59 |  |
| friends | not/little helpful | 163 (12.6) | 44.98 | 18.59 | 0.082 |
|  | quite/very helpful | 1,131 (87.4) | 42.28 | 18.49 |  |
| professional attitude/dissociation | not/little helpful | 122 (9.4) | 46.47 | 18.23 | 0.016* |
|  | quite/very helpful | 1,171 (90.6) | 42.23 | 18.49 |  |
| positive thinking | not/little helpful | 151 (11.7) | 46.44 | 18.26 | 0.007* |
|  | quite/very helpful | 1,135 (88.3) | 42.12 | 18.48 |  |
| hobbies | not/little helpful | 168 (13.1) | 48.33 | 20.26 | < 0.001** |
|  | quite/very helpful | 1,113 (86.9) | 41.78 | 18.08 |  |
| self-reflection | not/little helpful | 172 (13.3) | 45.57 | 18.01 | 0.023* |
|  | quite/very helpful | 1,117 (86.7) | 42.14 | 18.51 |  |
| self-care | not/little helpful | 175 (13.6) | 49.00 | 19.38 | < 0.001** |
|  | quite/very helpful | 1,116 (86.4) | 41.63 | 18.18 |  |
| physical activity | not/little helpful | 544 (42.3) | 43.73 | 18.72 | 0.060 |
|  | quite/very helpful | 743 (57.7) | 41.76 | 18.34 |  |
| religiosity/spirituality | not/little helpful | 685 (53.0) | 42.70 | 18.74 | 0.799 |
|  | quite/very helpful | 608 (47.0) | 42.44 | 18.17 |  |
| resilience | low/moderate | 629 (50.2) | 44.74 | 17.74 | < 0.001** |
|  | high | 623 (49.8) | 40.53 | 19.18 |  |

*Note.* Shown are valid percentages, M = mean, SD = standard-deviation, T-test for independent samples, analysis of variance or the chi-squared test were used, *p ≤ 0.05, **p ≤ 0.01
