# Supplementary material for: Health and intention to leave the profession of nursing - which individual, social and organisational resources buffer the impact of quantitative demands? A cross-sectional study
Source: BMC Palliat Care. 2020 Jun 17;19:83. doi: 10.1186/s12904-020-00589-y (PMC7298824; doi:10.1186/s12904-020-00589-y)
Supplement: Supplementary file 2 — Additional file 2: Table 2. Associations between the scale ‘burnout’ and covariates. [file 12904_2020_589_MOESM2_ESM.docx]

Additional Table 2: Associations between the scale ‘burnout’ and covariates

| **Variable** |  | **n** | **M** | **SD** | **p** |
| --- | --- | --- | --- | --- | --- |
| sex | male | 161 | 37.16 | 16.56 | 0.001** |
|  | female | 1,116 | 41.90 | 17.59 |  |
| age | ≤ 39 years | 341 | 41.47 | 17.72 | 0.977 |
|  | 40 – 49 years | 365 | 41.20 | 17.39 |  |
|  | ≥ 50 years | 579 | 41.37 | 17.65 |  |
| marital status | single | 314 | 41.83 | 17.60 | 0.278 |
|  | married | 720 | 40.73 | 17.55 |  |
|  | divorced/widowed | 233 | 42.73 | 17.66 |  |
| children in household | no | 706 | 41.25 | 17.74 | 0.634 |
|  | yes | 564 | 41.72 | 17.30 |  |
| graduation | without a school-leaving qualification/ secondary school leaving certificate/ other qualification | 71 | 39.34 | 19.28 | 0.357 |
|  | intermediate school-leaving certificate | 674 | 41.93 | 17.47 |  |
|  | qualification for university entrance | 534 | 40.88 | 17.42 |  |
| education | nursing assistant/ in training | 220 | 40.56 | 17.00 | 0.657 |
|  | geriatric nurse | 136 | 42.12 | 19.08 |  |
|  | nurse | 832 | 41.62 | 17.54 |  |
|  | university graduate | 95 | 39.83 | 17.12 |  |
| working area | SAPV | 336 | 40.54 | 17.56 | 0.008** |
|  | hospice | 536 | 40.25 | 17.711 |  |
|  | palliative unit | 439 | 43.56 | 17.36 |  |
| duration of nursing activities*** | in years | 1,283 |  |  | 0.012* |
| exercise of nursing procedures | no | 232 | 38.88 | 16.65 | 0.016* |
|  | yes | 1,067 | 41.94 | 17.75 |  |
| extent of employment | full-time job | 573 | 41.40 | 17.81 | 0.060 |
|  | ≥ 76 % | 181 | 43.46 | 17.89 |  |
|  | 51 - 75% | 315 | 42.11 | 17.80 |  |
|  | ≤ 50% | 221 | 38.91 | 16.35 |  |
| fund | publicly-owned | 336 | 42.42 | 17.34 | 0.345 |
|  | private | 208 | 41.84 | 18.86 |  |
|  | independent | 729 | 40.80 | 17.13 |  |

*Note.* Shown are valid percentages, M = mean, SD = standard deviation, T-test for independent samples or analysis of variance were used; *p ≤ 0.05, **p ≤ 0.01, ***duration of nursing activities: n = 1,283, r = 0.070, p = 0.012
