# Supplementary material for: Health and intention to leave the profession of nursing - which individual, social and organisational resources buffer the impact of quantitative demands? A cross-sectional study
Source: BMC Palliat Care. 2020 Jun 17;19:83. doi: 10.1186/s12904-020-00589-y (PMC7298824; doi:10.1186/s12904-020-00589-y)
Supplement: Supplementary file 3 — Additional file 3: Table 3. Associations between ‘intention to leave the profession’ and covariates. [file 12904_2020_589_MOESM3_ESM.docx]

Additional Table 3: Associations between ‘intention to leave the profession’ and covariates

| **Variables** |  |  | **Intention to leave** | |  |
| --- | --- | --- | --- | --- | --- |
|  |  | **n** | **no (n, %)** | **yes (n, %)** | **p** |
| sex |  | 1,277 |  |  | 0.656 |
|  | male |  | 98 (12.4) | 65 (13.3) |  |
|  | female |  | 690 (87.6) | 424 (86.7) |  |
| age |  | 1,285 |  |  | 0.401 |
|  | ≤ 39 years |  | 206 (25.9) | 135 (27.6) |  |
|  | 40 – 49 years |  | 237 (29.8) | 129 (26.3) |  |
|  | ≥ 50 years |  | 352 (44.3) | 226 (46.1) |  |
| marital status |  | 1,267 |  |  | 0.003** |
|  | single |  | 169 (21.6) | 146 (30.0) |  |
|  | married |  | 467 (59.8) | 252 (51.9) |  |
|  | divorced/widowed |  | 145 (18.6) | 88 (18.1) |  |
| children in household |  | 1,270 |  |  | < 0.001** |
|  | no |  | 402 (51.3) | 302 (62.1) |  |
|  | yes |  | 382 (48.7) | 184 (37.9) |  |
| graduation |  | 1,279 |  |  | 0.092 |
|  | without a school-leaving qualification/ secondary school leaving certificate/ other qualification |  | 46 (5.8) | 26 (5.3) |  |
|  | intermediate school-leaving certificate |  | 431 (54.5) | 238 (48.8) |  |
|  | qualification for university entrance |  | 314 (39.7) | 224 (45.9) |  |
| education |  | 1,284 |  |  | 0.026* |
|  | nursing assistant/ in training |  | 132 (16.7) | 88 (17.9) |  |
|  | geriatric nurse |  | 100 (12.6) | 36 (7.3) |  |
|  | nurse |  | 500 (63.1) | 332 (67.5) |  |
|  | university graduate |  | 60 (7.6) | 36 (7.3) |  |
| working area |  | 1,311 |  |  | < 0.001** |
|  | SAPV |  | 228 (28.1) | 109 (21.8) |  |
|  | hospice |  | 345 (42.5) | 190 (38.0) |  |
|  | palliative unit |  | 238 (29.3) | 201 (40.2) |  |
| duration of nursing activities*** |  |  |  |  | 0.001** |
| exercise of nursing procedures |  | 1,299 |  |  | 0.003** |
|  | no |  | 164 (20.4) | 69 (13.9) |  |
|  | yes |  | 639 (79.6) | 427 (86.1) |  |
| extent of employment |  | 1,291 |  |  | < 0.001** |
|  | full-time job |  | 378 (47.4) | 196 (39.8) |  |
|  | ≥ 76 % |  | 94 (11.8) | 87 (17.6) |  |
|  | 51 - 75% |  | 176 (22.1) | 140 (28.4) |  |
|  | ≤ 50% |  | 150 (18.8) | 70 (14.2) |  |
| fund |  | 1,273 |  |  | 0.042* |
|  | publicly-owned |  | 191 (24.2) | 146 (30.2) |  |
|  | private |  | 139 (17.6) | 70 (14.5) |  |
|  | independent |  | 460 (58.2) | 267 (55.3) |  |

*Note.* Shown are valid percentages, chi-squared test was used; *p ≤ 0.05, **p ≤ 0.01, ***duration of nursing activities: n = 1,283, B (SE) = 0,040, OR = 1.040, 95% CI [1.016-1.065], p = 0.001
