# Supplementary material for: Health and intention to leave the profession of nursing - which individual, social and organisational resources buffer the impact of quantitative demands? A cross-sectional study
Source: BMC Palliat Care. 2020 Jun 17;19:83. doi: 10.1186/s12904-020-00589-y (PMC7298824; doi:10.1186/s12904-020-00589-y)
Supplement: Supplementary file 4 — Additional file 4: Table 4. Coefficients of the moderated regression model for ‘self-rated health’ [file 12904_2020_589_MOESM4_ESM.docx]

Additional Table 4: Coefficients of the moderated regression model for ‘self-rated health’

|  |  | recognition through salary | | | | good working team | | | |
| --- | --- | --- | --- | --- | --- | --- | --- | --- | --- |
|  |  | b | SE | t | p | b | SE | t | p |
| (constant) |  | 78.68 [74.04, 83.323] | 2.367 | 33.24 | < 0.001 | 79.56 [74.87, 84.25] | 2.390 | 33.29 | < 0.001 |
| age | ≤ 39 years | 5.03 [2.76, 7.30] | 1.158 | 4.35 | < 0.001 | 4.67 [2.37, 6.97] | 1.173 | 3.98 | < 0.001 |
|  | 40 - 49 years | 2.63 [0.49, 4,76] | 1.090 | 2.41 | 0.016 | 2.41 [0.25, 4.58] | 1.105 | 2.185 | 0.029 |
|  | ≥ 50 years | Ref. |  |  |  | Ref. |  |  |  |
| sex | male | Ref. |  |  |  | Ref. |  |  |  |
|  | female | -3.03 [-5.74, -0.33] | 1.378 | -2.20 | 0.028 | 2.80 [-5.53, -0.07] | 1.392 | -2.01 | 0.044 |
| working area | SAPV | -1.95 [-4.90, 1.01] | 1.505 | -1.29 | 0.196 | - 1.36 [-4.32, 1.61] | 1.511 | -0.90 | 0.369 |
|  | hospice | -2.32 [-4.50, -0.15] | 1.108 | -2.09 | 0.037 | -1.37 [-3.56, 0.81] | 1.114 | -1.23 | 0.219 |
|  | palliative unit | Ref. |  |  |  | Ref. |  |  |  |
| extent of employment | full-time job | Ref. |  |  |  | Ref. |  |  |  |
|  | ≥ 76 % | 1.77 [-0.96, 4.47] | 1.377 | 1.28 | 0.199 | 1.51 [-1.22, 4.24] | 1.392 | 1.08 | 0.279 |
|  | 51 - 75% | 0.88 [-1.42, 3.17] | 1.169 | 0.75 | 0.454 | 0.80 [-1.52, 3.12] | 1.182 | 0.68 | 0.499 |
|  | ≤ 50% | 2.47 [-0.15, 5.08] | 1.332 | 1.85 | 0.064 | 1.51 [-1.12, 4.14] | 1.341 | 1.12 | 0.261 |
| duration of nursing activities |  | -0.31 [-0.50, -0.12] | 0.098 | -3.17 | 0.002 | -0.32 [-0.52, -0.13] | 0.099 | -3.25 | 0.001 |
| exercise of nursing procedures | no | Ref. |  |  |  | Ref. |  |  |  |
|  | yes | -2.66 [-5.76, 0.44] | 1.580 | -1.68 | 0.093 | -4.19 [-7.28, -1.09] | 1.579 | -2.65 | 0.008 |
| **independent variable - demand** |  |  |  |  |  |  |  |  |  |
| scale quantitative demands |  | -0.24 [-0.29, -0.18] | 0.026 | -8.92 | < 0.001 | -0.24 [-0.29, -0.19] | 0.026 | -9.29 | < 0.001 |
| **resource** |  |  |  |  |  |  |  |  |  |
| recognition through salary | do not agree | Ref. |  |  |  |  |  |  |  |
|  | agree | 6.54 [4.46, 8.63] | 1.063 | 6.16 | < 0.001 |  |  |  |  |
| good working team | do not agree |  |  |  |  | Ref. |  |  |  |
|  | agree |  |  |  |  | 2.71 [-1.67, 7.10] | 2.233 | 1.22 | 0.224 |
| **Interaction** |  |  |  |  |  |  |  |  |  |
| scale quantitative demands*recognition through salary |  | 0.18 [0.07, 0.28] | 0.055 | 3.21 | 0.001 |  |  |  |  |
| scale quantitative demands*good working team |  |  |  |  |  | 0.35 [0.11, 0.59] | 0.122 | 2.89 | 0.004 |

*Note.* Recognition through salary: R^2^ = 0.146; good working team: R^2^ = 0.121; Ref: Reference
