# Supplementary material for: Health and intention to leave the profession of nursing - which individual, social and organisational resources buffer the impact of quantitative demands? A cross-sectional study
Source: BMC Palliat Care. 2020 Jun 17;19:83. doi: 10.1186/s12904-020-00589-y (PMC7298824; doi:10.1186/s12904-020-00589-y)
Supplement: Supplementary file 5 — Additional file 5: Table 5. Coefficients of the moderated regression model for ‘burnout’ [file 12904_2020_589_MOESM5_ESM.docx]

Additional Table 5: Coefficients of the moderated regression model for ‘burnout’

|  |  | workplace commitment | | | | good working team | | | |
| --- | --- | --- | --- | --- | --- | --- | --- | --- | --- |
|  |  | b | SE | t | p | b | SE | t | p |
| (constant) |  | 31.25 [26.65, 35.85] | 2.343 | 13.34 | < 0.001 | 30.84 [26.23, 35.45] | 2.351 | 13.12 | < 0.001 |
| age | ≤ 39 years | 2.00 [-0.25, 4.24] | 1.145 | 1.74 | 0.082 | 2.45 [0.19, 4.71] | 1.151 | 2.13 | 0.034 |
|  | 40 - 49 years | 0.15 [-1.96, 2.27] | 1.080 | 0.14 | 0.886 | 0.66 [-1.47, 2.79] | 1.085 | 0.61 | 0.541 |
|  | ≥ 50 years | Ref. |  |  |  | Ref. |  |  |  |
| sex | male | Ref. |  |  |  | Ref. |  |  |  |
|  | female | 3.79 [1.11, 6.47] | 1.366 | 2.77 | 0.006 | 3.75 [1.06, 6.43] | 1.369 | 2.74 | 0.006 |
| working area | SAPV | 2.02 [-0.90, 4.94] | 1.489 | 1.35 | 0.176 | 1.78 [-1.13, 4.70] | 1.486 | 1.20 | 0.230 |
|  | hospice | 2.21 [0.06, 4.36] | 1.096 | 2.01 | 0.044 | 1.98 [-0.17, 4.12] | 1.092 | 1.81 | 0.071 |
|  | palliative unit | Ref. |  |  |  | Ref. |  |  |  |
| extent of employment | full-time job | Ref. |  |  |  | Ref. |  |  |  |
|  | ≥ 76 % | 2.99 [0.30, 5.68] | 1.371 | 2.18 | 0.029 | 3.04 [0.35, 5.73] | 1.372 | 2.22 | 0.027 |
|  | 51 - 75% | 0.15 [-2.12, 2.41] | 1.156 | 0.13 | 0.899 | 0.24 [-2.03, 2.51] | 1.158 | 0.21 | 0.834 |
|  | ≤ 50% | -1.49 [-4.08, 1.10] | 1.320 | -1.13 | 0.259 | -1.10 [-3.69, 1.48] | 1.318 | -0.84 | 0.402 |
| duration of nursing activities |  | 0.17 [-0.02, 0.37] | 0.098 | 1.78 | 0.075 | 0.20 [0.004, 0.39] | 0.098 | 2.00 | 0.045 |
| exercise of nursing procedures | no | Ref. |  |  |  | Ref. |  |  |  |
|  | yes | 4.04 [1.01, 7.07] | 1.545 | 2.61 | 0.009 | 4.25 [1.20, 7.29] | 1.551 | 2.74 | 0.006 |
| **independent variable - demand** |  |  |  |  |  |  |  |  |  |
| scale quantitative demands |  | 0.42 [0.37, 0.47] | 0.026 | 16.61 | < 0.001 | 0.43 [0.38, 0.48] | 0.026 | 16.76 | < 0.001 |
| **resource** |  |  |  |  |  |  |  |  |  |
| workplace commitment | value of scale 0-50 | -2.60 [-4.50, -0.70] | 0.968 | -2.69 | 0.007 |  |  |  |  |
|  | value of scale 51-100 | Ref. |  |  |  |  |  |  |  |
| good working team | do not agree |  |  |  |  | Ref. |  |  |  |
|  | agree |  |  |  |  | -4.75 [-9.03, -0.47] | 2.182 | -2.18 | 0.030 |
| **interaction** |  |  |  |  |  |  |  |  |  |
| scale quantitative demands* workplace commitment |  | -0.17 [-0.27, -0.07] | 0.052 | -3.22 | 0.001 |  |  |  |  |
| scale quantitative demands*good working team |  |  |  |  |  | -0.33 [-0.56, -0.10] | 0.118 | -2.77 | 0.006 |

*Note.* Workplace commitment: R^2^ = 0,225; good working team: R^2^ = 0,211; Ref.: Reference
