# Supplementary material for: Health and intention to leave the profession of nursing - which individual, social and organisational resources buffer the impact of quantitative demands? A cross-sectional study
Source: BMC Palliat Care. 2020 Jun 17;19:83. doi: 10.1186/s12904-020-00589-y (PMC7298824; doi:10.1186/s12904-020-00589-y)
Supplement: Supplementary file 6 — Additional file 6: Table 6. Coefficients of the moderated logistic regression of ‘intention to leave’ and resource ‘degree of freedom’. [file 12904_2020_589_MOESM6_ESM.docx]

Additional Table 6: Coefficients of the moderated logistic regression of ‘intention to leave’ and resource ‘degree of freedom’

|  |  | **b** | **SE** | **OR** | **p** |
| --- | --- | --- | --- | --- | --- |
| (constant) |  | -0.93 [-1.72, -0.26] | 0.373 | 0.37 [0.18, 0.77] | 0.008 |
| age | ≤ 39 years | 0.06 [-0.29, 0.42] | 0.181 | 1.07 [0.75, 1.52] | 0.725 |
|  | 40 - 49 years | -0.07 [-0.40, 0.25] | 0.165 | 0.93 [0.67, 1.28] | 0.653 |
|  | ≥ 50 years | Ref. |  |  |  |
| sex | male | Ref. |  |  |  |
|  | female | -0.28 [-0.67, 0.10] | 0.197 | 0.75 [0.51, 1.11] | 0.150 |
| working area | SAPV | 0,061 | 0,236 | 1,063 | 0.797 |
|  | hospice | 0,006 | 0,181 | 1,006 | 0.974 |
|  | palliative unit | Ref. |  |  |  |
| extent of employment | full-time job | Ref. |  |  |  |
|  | ≥ 76 % | 0.62 [0.23, 1.01] | 0.199 | 1.85 [1.25, 2.74] | 0.002 |
|  | 51 - 75% | 0.48 [0.14, 0.81] | 0.171 | 1.61 [1.15, 2.26] | 0.005 |
|  | ≤ 50% | 0.12 [-0.28, 0.52] | 0.204 | 1.13 [0.76, 1.69] | 0.546 |
| marital status | single | 0.41 [0.07, 0.75] | 0.174 | 1.51 [1.07, 2.12] | 0.018 |
|  | married | Ref. |  |  |  |
|  | divorced/  widowed | 0.11 [-0.24, 0.46] | 0.180 | 1.12 [0.79, 0.63] | 0.534 |
| children in household | no | Ref. |  |  |  |
|  | yes | -0.33 [-0.62, -0.04] | 0.148 | 0.72 [0.54, 0.96] | 0.024 |
| education | nursing assistant/ in training | 0.03 [-0.32, 0.38] | 0.178 | 1.03 [0.72, 1.46] | 0.881 |
|  | geriatric nurse | -0.56 [-1.03, -0.08] | 0.243 | 0.57 [0.36, 0.92] | 0.022 |
|  | nurse | Ref. |  |  |  |
|  | studies | -0.10 [-0.63, 0.44] | 0.273 | 0.91 [0.53, 1.55] | 0.719 |
| duration of nursing activities |  | 0.04 [0.01, 0.07] | 0.014 | 1.04 [1.01, 1.07] | 0.004 |
| exercise of nursing procedures | no | Ref. |  |  |  |
|  | yes | 0.24 [-0.23, 0.72] | 0.244 | 1,28 [0.79, 2.06] | 0.315 |
| fund | Publicly owned | 0.11 [-0.22, 0.45] | 0.172 | 1.12 [0.80, 1.57] | 0.506 |
|  | private | 0.01 [-0.39, 0.40] | 0.201 | 1.01 [0.68, 1.50] | 0.968 |
|  | independent | Ref. |  |  |  |
| **independent variable - demand** |  |  |  |  |  |
| scale quantitative demands |  | 0.02 [0.02, 0.03] | 0.004 | 1.02 [1.02, 1.03] | < 0.001 |
| **resource** |  |  |  |  |  |
| scale degree of freedom |  | -0.01 [-0.02,  -0.002] | 0.004 | 0.99 [0.98, 0.998] | 0.013 |
| **interaction** |  |  |  |  |  |
| scale quantitative demands * scale degree of freedom |  | -0.0005 [-0.0008,  -0.0001 | 0.0002 | 0.9995 [0.9992, 0.9999] | 0.014 |

*Note.* R^2^ (Nagelkerke) = 0,154; OR = Odds Ratio; Ref.: Reference
