# Supplementary material for: Health and intention to leave the profession of nursing - which individual, social and organisational resources buffer the impact of quantitative demands? A cross-sectional study
Source: BMC Palliat Care. 2020 Jun 17;19:83. doi: 10.1186/s12904-020-00589-y (PMC7298824; doi:10.1186/s12904-020-00589-y)
Supplement: Supplementary file 7 — Additional file 7: Table 7. Coefficients of the moderated logistic regression of ‘intention to leave’ and ‘meeting relatives after death’. [file 12904_2020_589_MOESM7_ESM.docx]

Additional Table 7: Coefficients of the moderated logistic regression of ‘intention to leave’ and ‘meeting relatives after death’

|  |  | **b** | **SE** | **OR** | **p** |
| --- | --- | --- | --- | --- | --- |
| (constant) |  | -1.04 [-1.77, -0.31] | 0.372 | 0.35 [0.17, 0.73] | 0.005 |
| age | ≤ 39 years | 0.05 [-0.30, 0.41] | 0.181 | 1.05 [0.74, 1.50] | 0.769 |
|  | 40 - 49 years | -0.12 [-0.45, 0.20] | 0.166 | 0.88 [0.64, 1.22] | 0.456 |
|  | ≥ 50 years | Ref. |  |  |  |
| sex | male | Ref. |  |  |  |
|  | female | -0.20 [-0.59, 0.19] | 0.199 | 0.82 [0.56, 1.21] | 0.321 |
| working area | SAPV | -0.06 [-0.52, 0.39] | 0.231 | 0.94 [0.60, 1.48] | 0.786 |
|  | hospice | -0.08 [-0.43, 0.27] | 0.179 | 0.92 [0.65, 1.31] | 0.650 |
|  | palliative unit | Ref. |  |  |  |
| extent of employment | full-time job | Ref. |  |  |  |
|  | ≥ 76 % | 0.65 [0.26, 1.04] | 0.198 | 1.92 [1.30, 2.83] | 0.001 |
|  | 51 - 75% | 0.46 [0.12, 0.80] | 0.171 | 1.58 [1.13, 2.22] | 0.007 |
|  | ≤ 50% | 0.18 [-0.22, 0.57] | 0.202 | 1.19 [0.80, 1.78] | 0.381 |
| marital status | single | 0.41 [0.07, 0.75] | 0.174 | 1.51 [1.08, 2.13] | 0.017 |
|  | married | Ref. |  |  |  |
|  | divorced/ widowed | 0.11 [-0.24, 0.46] | 0.178 | 1.11 [0.79, 1.58] | 0.546 |
| children in household | no | Ref. |  |  |  |
|  | yes | -0.32 [-0.60, -0.03] | 0.147 | 0.73 [0.55, 0.97] | 0.031 |
| education | nursing assistant/ in training | 0.04 [-0.31, 0.39] | 0.179 | 1.04 [0.74, 1.48] | 0.810 |
|  | geriatric nurse | -0.48 [-0.95, -0.01] | 0.242 | 0.62 [0.39, 0.995] | 0.048 |
|  | nurse | Ref. |  |  |  |
|  | studies | -0.15 [-0.67, 0.37] | 0.267 | 0.86 [0.51, 1.45] | 0.577 |
| duration of nursing activities |  | 0.04 [0.01, 0.07] | 0.014 | 1.04 [1.11, 1.07] | 0.007 |
| exercise of nursing procedures | no | Ref. |  |  |  |
|  | yes | 0.39 [-0.08, 0.85] | 0.237 | 1.47 [0.92, 2.34] | 0.103 |
| fund | publicly-owned | 0.08 [-0.26, 0.441] | 0.172 | 1.08 [0.77, 1.51] | 0.662 |
|  | private | -0.01 [-0.40, 0.39] | 0.200 | 0.99 [0.67, 1.47] | 0.970 |
|  | independent | Ref. |  |  |  |
| **independent variable - demand** |  |  |  |  |  |
| scale quantitative demands |  | 0.03 [0.02, 0.04] | 0.004 | 1.03 [1.02, 1.04] | < 0.001 |
| **resource** |  |  |  |  |  |
| meeting relatives after death | not, little | Ref. |  |  |  |
|  | quite, very | -0.34 [-0.60, -0.08] | 0.134 | 0.71 [0.55, 0.93] | 0.011 |
| **interaction** |  |  |  |  |  |
| scale quantitative demands * meeting relatives after death |  | -0.02 [-0.03,  -0.004] | 0.007 | 0.98 [0.97, 0.996] | 0.012 |

*Note.* R^2^ (Nagelkerke) = 0,150; OR = Odds Ratio; Ref.: Reference
