# Supplementary material for: Health and intention to leave the profession of nursing - which individual, social and organisational resources buffer the impact of quantitative demands? A cross-sectional study
Source: BMC Palliat Care. 2020 Jun 17;19:83. doi: 10.1186/s12904-020-00589-y (PMC7298824; doi:10.1186/s12904-020-00589-y)
Supplement: Supplementary file 8 — Additional file 8: Table 8. Coefficients of the moderated logistic regression of ‘intention to leave’ and resource ‘recognition from supervisor’. [file 12904_2020_589_MOESM8_ESM.docx]

Additional Table 8: Coefficients of the moderated logistic regression of ‘intention to leave’ and resource ‘recognition from supervisor’

|  |  | **b** | **SE** | **OR** | **p** |
| --- | --- | --- | --- | --- | --- |
| (constant) |  | -1,27 [-2.01, -0.52] | 0.380 | 0.28 [0.13, 0.59] | 0.001 |
| age | ≤ 39 years | 0.07 [-0.29, 0.43] | 0.184 | 1.07 [0.74, 1.53] | 0.720 |
|  | 40 - 49 years | -0.13 [-0.46, 0.21] | 0.169 | 0.88 [0.63, 1.23] | 0.457 |
|  | ≥ 50 years | Ref. |  |  |  |
| sex | male | Ref. |  |  |  |
|  | female | -0.22 [-0.62, 0.18] | 0.202 | 0.80 [0.54, 1.19] | 0.268 |
| working area | SAPV | 0.08 [-0.38, 0.55] | 0.237 | 1.09 [0.68, 1.73] | 0.727 |
|  | hospice | 0.04 [-0.32, 0.40] | 0.182 | 1.04 [0.73, 1.49] | 0.832 |
|  | palliative unit | Ref. |  |  |  |
| extent of employment | full-time job | Ref. |  |  |  |
|  | ≥ 76 % | 0.74 [0.34, 1.13] | 0.202 | 2.09 [1.41, 3.11] | < 0.001 |
|  | 51 - 75% | 0.60 [0.25, 0.94] | 0.176 | 1.82 [1.29, 2.57] | 0.001 |
|  | ≤ 50% | 0.23 [-0.18, 0.63] | 0.207 | 1.26 [0.84, 1.89] | 0.271 |
| marital status | single | 0.47 [0.12, 0.82] | 0.178 | 1.60 [1.13, 2.27] | 0.008 |
|  | married | Ref. |  |  |  |
|  | divorced/ widowed | 0.14 [-0.22, 0.50] | 0.183 | 1.15 [0.80, 1.64] | 0.446 |
| children in household | no | Ref. |  |  |  |
|  | yes | -0.29 [-0.59, 0.001] | 0.150 | 0.75 [0.56, 1.00] | 0.051 |
| education | nursing assistant/ in training | 0.03 [-0.33, 0.39] | 0.183 | 1.03 [0.72, 1.48] | 0.863 |
|  | geriatric nurse | -0.63 [-1.12, -0.14] | 0.250 | 0.53 [0.33, 0.87] | 0.011 |
|  | nurse | Ref. |  |  |  |
|  | studies | -0.14 [-0.68, 0.40] | 0.274 | 0.87 [0.51, 1.48] | 0.604 |
| duration of nursing activities |  | 0.05 [0.02, 0.08] | 0.015 | 1.05 [1.02, 1.08] | 0.001 |
| exercise of nursing procedures | no | Ref. |  |  |  |
|  | yes | 0.35 [-0.13, 0.82] | 0.243 | 1.41 [0.88, 2.28] | 0.156 |
| fund | publicly-owned | 0.15 [-0.17, 0.49] | 0.174 | 1.16 [0.83, 1.63] | 0.389 |
|  | private | -0.32 [-0.44, 0.37] | 0.206 | 0.97 [0.65, 1.45] | 0.875 |
|  | independent | Ref. |  |  |  |
| **independent variable - demand** |  |  |  |  |  |
| scale quantitative demands |  | 0.02 [0.01, 0.03] | 0.004 | 1.02 [1.01, 1.03] | < 0.001 |
| **resource** |  |  |  |  |  |
| recognition from supervisor | do not agree | Ref. |  |  |  |
|  | agree | -0.89 [-1.18, 0.60] | 0.149 | 0.41 [0.31, 0.55] | < 0.001 |
| **interaction** |  |  |  |  |  |
| scale quantitative demands * recognition from supervisor |  | -0.02 [-0.04, -0.01] | 0.008 | 0.98 [0.96, 0.99] | 0.007 |

*Note.* R^2^ (Nagelkerke) = 0,191; OR = Odds Ratio; Ref.: Reference
