# Supplementary material for: Health and intention to leave the profession of nursing - which individual, social and organisational resources buffer the impact of quantitative demands? A cross-sectional study
Source: BMC Palliat Care. 2020 Jun 17;19:83. doi: 10.1186/s12904-020-00589-y (PMC7298824; doi:10.1186/s12904-020-00589-y)
Supplement: Supplementary file 9 — Additional file 9: Table 9. Coefficients of the moderated logistic regression of ‘intention to leave’ and resource ‘possibilities for development’. [file 12904_2020_589_MOESM9_ESM.docx]

Additional Table 9: Coefficients of the moderated logistic regression of ‘intention to leave’ and resource ‘possibilities for development’

|  |  | **B** | **SE** | **OR** | **p** |
| --- | --- | --- | --- | --- | --- |
| (constant) |  | -1.04 [-1.77, -0.31] | 0.373 | 0.35 [0.17, 0.74] | 0.005 |
| age | ≤ 39 years | 0.05 [-0.30, 0.41] | 0.181 | 1.05 [0.74, 1.50] | 0.770 |
|  | 40 - 49 years | -0.10 [-0.43, 0.22] | 0.165 | 0.90 [0.65, 1.25] | 0.534 |
|  | ≥ 50 years | Ref. |  |  |  |
| sex | male | Ref. |  |  |  |
|  | female | -0.23 [-0.62, 0.16] | 0.199 | 0.79 [0.54, 1.17] | 0.249 |
| working area | SAPV | 0.04 [-0.42, 0.50] | 0.234 | 1.04 [0.66, 1.64] | 0.869 |
|  | hospice | 0.03 [-0.32, 0.38] | 0.181 | 1.03 [0.72, 1.47] | 0.868 |
|  | palliative unit | Ref. |  |  |  |
| extent of employment | full-time job | Ref. |  |  |  |
|  | ≥ 76 % | 0.68 [0.29, 1.07] | 0.199 | 1.97 [1.33, 2.91] | 0.001 |
|  | 51 - 75% | 0.50 [0.16, 0.83] | 0.172 | 1.65 [1.18, 2.30] | 0.004 |
|  | ≤ 50% | 0.17 [-0.23, 0.56] | 0.203 | 1.18 [0.79, 1.76] | 0.410 |
| marital status | single | 0.38 [0.03, 0.72] | 0.175 | 1.46 [1.04, 2.05] | 0.031 |
|  | married | Ref. |  |  |  |
|  | divorced/ widowed | 0.10 [-0.25, 0.45] | 0.179 | 1.10 [0.78, 1.57] | 0.585 |
| children in household | no | Ref. |  |  |  |
|  | yes | -0.31 [-0.59, -0.02] | 0.147 | 0.74 [0.55, 0.98] | 0.038 |
| education | nursing assistant/ in training | 0.07 [-0.28, 0.42] | 0.178 | 1.07 [0.75, 1.52] | 0.703 |
|  | geriatric nurse | -0.50 [-0,97, -0.03] | 0.242 | 0.61 [0.38, 0.97] | 0.039 |
|  | nurse | Ref. |  |  |  |
|  | studies | -0.08 [-0.60, 0.45] | 0.267 | 0.93 [0.55, 1.56] | 0.775 |
| duration of nursing activities |  | 0.04 [0.01, 0.07] | 0.014 | 1.04 [1.01, 1.07] | 0.005 |
| exercise of nursing procedures | no | Ref. |  |  |  |
|  | yes | 0.29 [-0.18, 0.76] | 0.239 | 1.34 [0.84, 2.14] | 0.226 |
| fund | publicly-owned | 0.09 [-0.25, 0.43] | 0.173 | 1.09 [0.78, 1.53] | 0.616 |
|  | private | 0.05 [-0.35, 0.44] | 0.200 | 1.05 [0.71, 1.55] | 0.818 |
|  | independent | Ref. |  |  |  |
| **independent variable - demand** |  |  |  |  |  |
| scale quantitative demands |  | 0.03 [0.02, 0.04] | 0.004 | 1.03 [1.02, 1.04] | < 0.001 |
| **resource** |  |  |  |  |  |
| scale possibilities for development |  | -0.02 [-0.03, -0.01] | 0.005 | 0.98 [0.97, 0.99] | < 0.001 |
| **interaction** |  |  |  |  |  |
| scale quantitative demands * scale possibilities for development |  | -0.0005 [-0.001, 0.000] | 0,0002 | 0.9995 [0.9992, 1.0] | 0.036 |

*Note.* R^2^ (Nagelkerke) = 0,163; OR = Odds Ratio; Ref.: Reference
